# Surgical removal of superficial peritoneal endometriosis for managing women with chronic pelvic pain: time for a rethink?

**DOI:** 10.1111/1471-0528.15894

**Published:** 2019-08-16

**Authors:** AW Horne, J Daniels, L Hummelshoj, E Cox, KG Cooper

**Affiliations:** ^1^ MRC Centre for Reproductive Health University of Edinburgh Edinburgh UK; ^2^ Department of Clinical Trials Nottingham Clinical Trials Unit University of Nottingham Nottingham UK; ^3^ Endometriosis.org London UK; ^4^ Endometriosis London UK; ^5^ Department of Obstetrics and Gynaecology Aberdeen Royal Infirmary Aberdeen UK

Endometriosis is a potentially life‐altering, estrogen‐dependent condition which is associated with chronic pelvic pain. It affects an estimated 176 million women worldwide, making it as common as diabetes mellitus (DM).^1^ The socio‐economic burden of endometriosis in the UK is in excess of £8.2 billion per year, with average worldwide costs amounting to around £8,500 per woman per year (similar again to DM).^2^ Evidence suggests that women with endometriosis are at higher risk of infertility, ovarian and breast cancer, melanoma, asthma, and some autoimmune, cardiovascular, and atopic diseases.^3^ A diagnosis of endometriosis should be considered when a woman presents with chronic pelvic pain and, as there are no accurate noninvasive biomarkers of endometriosis, the diagnosis generally necessitates a laparoscopy.^1^, ^4^, ^5^

Endometriosis is defined by the presence of endometrial‐like tissue (‘lesions’) outside the uterus. Three subtypes of endometriosis have been described: superficial peritoneal, ovarian (endometrioma or ‘chocolate cysts’), and deep.^1^ Superficial peritoneal endometriosis (SPE) is the most common and accounts for ~80% of all endometriosis. However, SPE is by no means a homogeneous condition—phenotypically its location and its extent varies, and it can co‐exist with the other subtypes, as well as with adenomyosis. In addition, despite pain being the cardinal symptom of endometriosis, the underlying biological mechanisms of endometriosis‐associated pain are not known for any subtype. The natural history of the disease is uncertain, e.g. it is not known whether SPE can progress to become another subtype, regress spontaneously, or whether disease progression (or lack of treatment) can lead to problems with infertility. Furthermore, there is poor correlation between pain severity and the amount, location, and subtype of the endometriotic lesions.^1^

The management options in national and international endometriosis guidelines for women with all endometriosis subtypes and condition‐associated symptoms include surgical removal of lesions and medical treatment with ovarian suppressive drugs.^1^, ^4^, ^5^ The guidelines suggest that clinicians consider a ‘see and treat’ approach when SPE is identified at a diagnostic laparoscopy.^4^, ^5^ Surgical removal’ requires appropriate surgical expertise and involves laparoscopic excision and/or ablation of the endometriotic lesions. Complete surgical removal is dependent on accurate recognition of the condition, including its extent and distribution, in addition to having the requisite skills safely and proficiently to excise or ablate the disease. These are skills that may be beyond those not specifically trained in this area, and there is perhaps an argument for diagnostic laparoscopy for pelvic pain to be undertaken only by those who are trained.

The evidence behind these national and international endometriosis guideline recommendations are largely summarised in a meta‐analysis of available data that concludes that laparoscopic treatment improves condition‐associated pain (cited as ‘better’ or ‘improved’) compared with diagnostic laparoscopy alone at 6 months (odds ratio [OR] 6.58, 95% CI 3.31–13.10)’.^6^ However, this statement is based on data from just three randomised controlled trials (RCT), a total of only 171 participants with all three different subtypes of endometriosis, the use of multiple treatment modalities to remove the lesions, and data of ‘moderate quality’ when scored using GRADE (a recognised systematic and explicit approach to making judgements about the quality of evidence and strength of recommendations). Furthermore, only one RCT included in the analysis (with only 69 participants) has follow‐up data to 12 months showing benefit of surgery (OR 10.00, 95% CI 3.21–31.17). Using GRADE, this represents ‘low quality evidence’. Thus, there is little evidence to demonstrate whether surgical removal of isolated SPE at diagnostic laparoscopy improves overall symptoms and quality of life. Indeed, it has been proposed that SPE may not be responsible for pain, and that it may be due to other overlooked causes (e.g. irritable bowel syndrome, bladder pain syndrome, musculoskeletal disorders, somatic symptom disorder). Treatment for these other conditions may be delayed by concomitant surgical treatment at the time of diagnostic laparoscopy because recovery may be prolonged and due to subsequent time lapses while symptomatic outcomes are evaluated.^7^

Consequently, the UK National Institute of Clinical Excellence (NICE) Endometriosis Guideline recommends further research into the effectiveness of laparoscopic treatment of SPE to manage endometriosis‐associated pain.^5^ This research recommendation is supported by the outcome of the James Lind Alliance Priority Setting Partnership Initiative for Endometriosis, established to identify the key research questions prioritised by both women with endometriosis and the healthcare practitioners involved in their care.^8^ We also believe that it is important to establish whether treating SPE in isolation by surgery is effective. Diagnostic laparoscopies for suspected endometriosis form a large part of the workload in general gynaecology, utilising resources at considerable cost to health services. Scottish data (population 5.4 million; http://www.isdscotland.org) indicate that over 83 000 diagnostic laparoscopies were performed in women from 1981 to 2010, of which ~90% were for the investigation of chronic pelvic pain, with 42 092 women receiving a diagnosis of endometriosis, of which an estimated 33 700 had SPE.^9^ In all, 62% of the women studied had a repeat operation following initial surgical diagnosis and 25% underwent more than three subsequent procedures, suggesting ineffectiveness of the primary surgical procedure. These observations are consistent with the worldwide persistence and recurrence rates of endometriosis after surgery: 21.5% within 2 years, and 40–50% after 5 years.^10^ Furthermore, there is concern over the increasingly wide range of non‐evidence‐based surgical approaches (e.g. stripping of the entire peritoneum) or use of novel energy modalities (e.g. helium beam) for treating SPE.

We, therefore, believe that a large, high‐quality, randomised clinical trial is urgently needed to determine whether surgical excision/ablation is of clinical benefit to women with chronic pelvic pain where the only finding is SPE. If the trial demonstrates that surgical removal of lesions at the time of laparoscopic diagnosis is effective, we anticipate that a sufficiently powered trial could identify the subgroups of women with SPE who will derive most benefit from surgery and determine which (if any) surgical approach to remove the endometriosis lesions is best. If the trial demonstrates that surgical removal of lesions is not effective for women with SPE, it is possible that women with chronic pelvic pain may ultimately choose to avoid a diagnostic laparoscopy and assume a ‘working diagnosis’ of SPE, in particular if their pelvic imaging does not reveal any pathology. These women could then opt for early pain management (e.g. analgesics, hormone treatments, neuromodulator drugs, physiotherapy, and psychological approaches) and potentially avoid unnecessary repeated surgical procedures.^10^ Like surgery for SPE, we acknowledge that some of these medical treatments also require further research to determine whether they are truly effective for the management of chronic pelvic pain, and so we also urgently need trials to address these uncertainties.^4^, ^5^ However, it is conceivable that future research could demonstrate that surgery for SPE in isolation is not only ineffective but aggravates the symptoms of pain, or even causes harm. There is increasing awareness of the problem of chronic postsurgical pain (CPSP), which occurs in ~20% of patients at 3–6 months, to the extent that 2017 was the International Association of Pain (IASP) Global Year Against Pain After Surgery (http://www.iasp-pain.org/GlobalYear). The factors identified as most predictive of CPSP are all prevalent in women with endometriosis.^11^, ^12^

In conclusion, we believe that it is crucial for policy makers, funding bodies, researchers, clinicians, and women with endometriosis to work together in a ‘precision medicine ecosystem’ to build a knowledge base that can determine whether SPE is better suited to surgical, multimodal or conservative treatment, to guide and improve individualised patient care.

## Source of review

We searched PubMed, Embase, NHS‐CRD (including DARE, NHS‐EED, HTA), and the Cochrane Library from their inception to June 2019 to identify published studies investigating effectiveness of surgery for the management of women with chronic pelvic pain associated with superficial peritoneal endometriosis.

## Disclosure of interests

Andrew Horne receives grant funding from the NIHR, MRC, Chief Scientist's Office, Wellcome Trust, Wellbeing of Women, Ferring, and Roche. He has received honoraria for consultancy for Ferring, Roche Diagnostics, Nordic Pharma, and Abbvie. Lone Hummelshoj has received honoraria for advice to AbbVie. Kevin Cooper, Jane Daniels, and Emma Cox have no conflicts of interest to declare. Completed disclosure of interests forms are available to view online as Supporting information.

## Contribution to authorship

All authors had an equal role in carrying out, analysing the data for and writing up this commentary.

## Details of ethics approval

Ethical approval was not required.

## Funding

This work was supported by funding from an MRC Centre Grant (MR/N022556/1).
